# Prevalence and clinical significance of antinuclear antibodies in Iranian women with unexplained recurrent miscarriage

**Published:** 2014-03

**Authors:** Morteza Molazadeh, Hadi Karimzadeh, Mohammad R Azizi

**Affiliations:** 1 *Department of Immunodiagnosis, Erythron Clinical Lab, Isfahan, Iran.*; 2 *Department of Rheumatology, Al-Zahra Hospital, Isfahan, Iran.*; 3 *Department of Immunology, Fertility and Infertility Center, Isfahan, Iran.*

**Keywords:** *Recurrent miscarriage*, *Antinuclear antibodies*, *Indirect immunofluorescence*

## Abstract

**Background:** Antinuclear antibodies (ANAs) in women with recurrent miscarriage have been reported. The presence of moderate to high titers of these antibodies represents an autoimmune condition that can endanger the health of the fetus in pregnant women.

**Objective:** In this study, we evaluated the prevalence of ANAs in Iranian women with a history of two or more unexplained abortion.

**Materials and Methods: **560 women with unexplained recurrent miscarriage and 560 healthy controls accounted for this study over a period of 13 months. ANAs were detected by indirect immunofluorescence technique.

**Results: **ANAs were detected in 74 of 560 (13.21%) patient with recurrent miscarriage, and in only 5 of 560 (0.9%) controls (p<0.001). ANA positivity was generally found with low-positive results (1.40-1.80) in about 38% of positive cases, whereas moderate titres (1.160-1.320) and high titres (>1.640) were seen in about 46% and 16% of cases respectively. Finally evaluating of microscopic ANA patterns revealed that about half of positive cases had antibodies against DNA- histone complex, associated with systemic lupus erythematosus disease.

**Conclusion:** Antinuclear antibodies are not uncommon in women with unexplained recurrent miscarriage, suggesting the possible role of an autoimmune disorder on abortion, at least in a subgroup of patients.

## Introduction

Recurrent Miscarriage (RM), defined as two or more abortion before 20 weeks pregnancy, affects 3% of all couples (1). Current diagnostic procedures identify etiological factors, such as translocations, immunologic factors, endocrine disorders and uterine abnormalities in 50% of these couples. The other 50% are diagnosed as couples with unexplained RM (2). 

Many researchers have been conducted to identify the underlying mechanisms of RM, and accumulating evidence reveals that an immunologic mechanism is involved in some miscarriage. However, several studies of the immune interactions at the feto-maternal interface and genetic-epidemiologic studies document an immunological background for many RM cases (3). Several autoantibodies, manufactured by the immune system directed against one or more of the individual’s own proteins have been investigated as possible influences on reproductive success and failure. Autoantibodies may persist for many years in the circulation as a marker of a prior autoimmune attack, but their presence does not necessarily indicate a current disease process. The anti-phospholipid antibodies such as lupus anticoagulant, anti-cardiolipin and anti- β2 glycoprotein are associated with RM or as possible factors involved in infertility (4). 

Antibodies to thyroid antigens, such as anti-thyroglobulin and anti-thyroid peroxidise, antibodies to nuclear antigens, anti-laminin, anti-prothrombin antibodies and anti-sacchromyces cerevisiae antibodies, have also been implicated in pregnancy complications (5-7). Antinuclear antibodies (ANAs) are as specific class of autoantibodies that have the capability of binding and destroying certain structures within the nucleus of the cells (8). Presently the ANAs have been categorized in two main groups; the first includes autoantibodies to DNA and histones and the second one consists autoantibodies to extractable nuclear antigens that are including autoantibodies to Smith antigen, ribonucleoproteins, SSA/Ro, SSB/La, Scl-70, Jo-1 (9). 

Indirect immunofluorescence technique using Hep-2 substrate has been the standard method for detecting these autoantibodies where can be present in a number of immunologic diseases, including systemic lupus erythematosus (SLE), progressive systemic sclerosis, Sjogren's syndrome, scleroderma polymyositis, dermatomyositis and in persons taking hydralazine and procainamide or isoniazid. In addition, ANAs are present in some normal individuals or those who have collagen vascular diseases (10).

In pregnant women the presence of ANAs indicates there may be an underlying autoimmune process that affects the development of the placenta and can lead to early pregnancy loss. Patients with unexplained recurrent abortion often have autoantibodies in blood as antiphospholipid antibodies and antinuclear antibodies (11). In the present study, we evaluated the incidence and importance of antinuclear antibodies in women with unexplained recurrent miscarriage.

## Materials and methods

**Study population**

This is a prospective observational study where all consecutive cases of unexplained RM were included between February 2012 and March 2013. 560 women with history of two or more consecutive unexplained RM in the first trimester were followed up sequentially in Isfahan Infertility Center (Isfahan, Iran), accounted for this study over a period of 13 months. The selected patients belonged to the age group of 22-35 and they were thoroughly investigated for all baseline blood parameters, and other infectious diseases including metabolic diseases. 

All other pathologic factors were ruled out except ANAs. Those patients, who showed negative for the above tests, were selected for the next step of ANAs screening test. An additional group including 560 healthy, age- and sex-matched controls was assessed in order to compare the frequency of ANA positivity in patients with unexplained RM vs. a sample of normal subjects. These controls had no evidence of inflammatory conditions.

**Indirect imunofluorescence**

For this purpose blood samples were collected from the patients then allowed to clot. After 30 min clotted samples were centrifuged at 4000 rpm for 10 min and purified serum was collected from the samples and kept in -20^o^C until future evaluation. ANAs were detected by indirect immunofluorescence technique on HEp-2 cells ANA kit (Euroimmune AG Company, Germany). ANA positivity was defined as a titre ≥1.40. A low positive test was defined as titres of 1.40-1.80, a moderate positive test was considered with ANA titres of 1.160-1.320, and a high positive test was considered with a titre of ANA ≥1.640. In addition to, ANA microscopic patterns were investigated for determination of type of anti-nuclear antibodies (anti-DNA, anti-histone and extractable nuclear antigens). 

**Statistical analysis**

Data were expressed as mean±SD. Comparison between patient and control groups was performed using unpaired t-test for continuous variables and a chi-square analysis for categorical variables. Statistical analysis was performed using SPSS 13.0 software. Odd ratios were given with 95% confidence intervals (CI). P<0.05 was considered to show statistical significance. 

## Results

**ANA titre**

**Figure 1:**
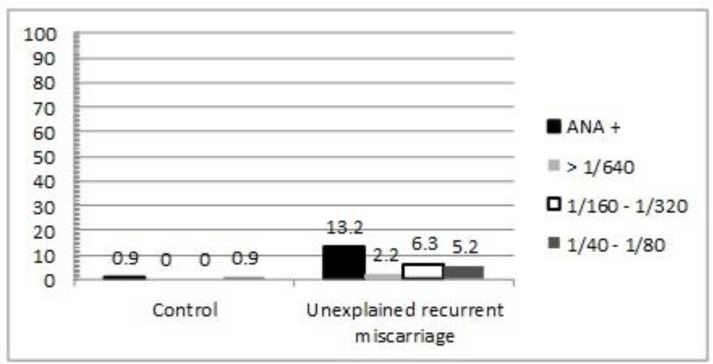
Relative frequencies of ANA positivity in patients with unexplained recurrent miscarriage and health age- and sex-matched controls (control vs. patients, respectively).

ANAs were detected in 74 of 560 (13.2%) unexplained RM. A positive ANA titre was recorded in only 5 of 560 (0.9%) healthy controls (p<0.001 vs. patients with unexplained RM, see Figure 1). In all cases normal subjects had a low titre (1.40-1.80). Among patients with unexplained RM, a low-positive titre of ANA (1.40-1.80) was recorded in 28 patients (5.2%), while a moderate-positive titre (1.160-1.320) was observed in 7 patients (6.3%). In 12 patients (2.2%) High-positive titres (≥1/640) were recorded (Figure 1).

**Microscopic patterns**

**Figure 2:**
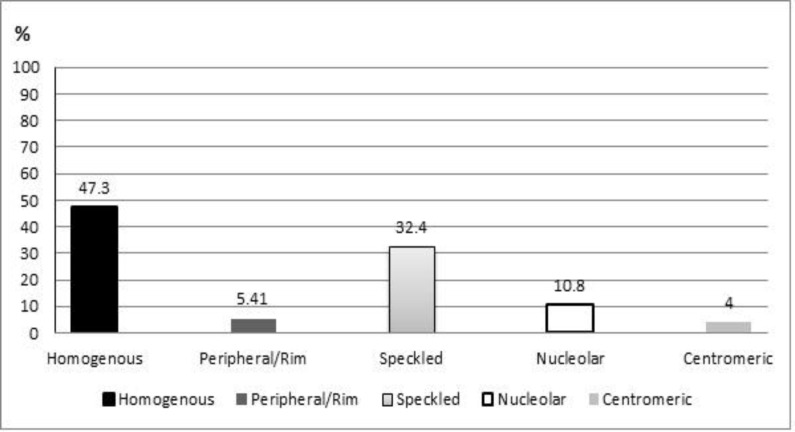
Relative frequencies of ANA patterns in patients with unexplained recurrent miscarriage

**Figure 3:**
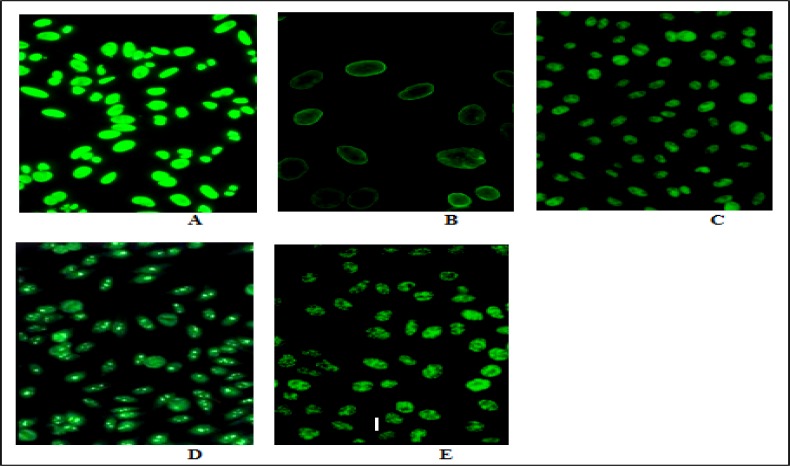
ANA patterns in patients with unexplained recurrent miscarriage by immunofluorescence microscopy.

Evaluating of ANA patterns by fluorescent microscopy revealed that 35 of 74 positive patients in unexplained RM group had homogenous pattern (47.3%), while 4 patients had peripheral/rim pattern (5.4%), 24 patients had speckled pattern (32.4%), 8 patients had nucleolar pattern (10.8%) and 3 patients (4%) had centromic pattern (see Figure 2). These patterns are illustrated in Figure 3.

Dark gray bars: ANA 1.40-1.80, p<0.001;

White bars: ANA 1.60-1.320, p<0.001

Grey bars: ANA ≥1.640, p<0.001;

Back bars: overall ANA positivity, p<0.001

A: Homogenous

B: Peripheral/rim

C: Speckled

D: Nuclear

E: Centromeric

## Discussion

Immunological responses could be the cause in many cases of infertility and miscarriage. Some immunological reasons that contribute to infertility are reproductive autoimmune failure syndrome, the presence of anti-phospholipid antibodies, and antinuclear antibodies (12). The mechanism by which ANAs cause pregnancy loss is not known well but based on a hypothesis antinuclear antibodies cause an inflammation in the uterus that does not allow it to be a suitable host for implantation of the embryo. Natural killer cells misinterpret the fetal cells as cancer cells and attack them (13). An individual that presents with reproductive autoimmune failure syndrome has unexplained infertility, endometriosis, and repetitive miscarriages due to elevated levels of antinuclear antibodies circulating (14).

Cubillos *et al* found that the incidence of ANAs was 31.8% in patients with a history of miscarriages (110 patients), but only 5.7% in 35 healthy patients with proven fertility and no history of pregnancy loss or autoimmune disease (15). Mavragani and et al shown that In women with autoimmune disorders, a history of recurrent pregnancy loss is independently associated with reactivity against antinuclear antibodies and also with the presence of antithyroglobulin antibodies (16).

In several studies, a high prevalence of low-titre ANA has been reported in the sera of patients with both explained and unexplained pregnancy losses. However, the significance of these findings is still unclear. Shoenfeld and coworkers were found antinuclear antibodies with a higher prevalence in patients with autoimmune disease. However, they were not found to occur with a higher prevalence in patients with infertility or recurrent pregnancy loss (17). ANA titres are important in the interpretation of the test but fluctuations in their titres have little clinical relevance in autoimmune disease. In one study of 125 cases with a positive ANA but no other evidence of connective tissue disease, titres greater than 1.40 were seen in 32%, greater than 1.80 were seen in 13%, and greater than 1.320 were only seen in 3% of patients (18). While the presence of high titres of antibodies (≥1.640) should arouse suspicion of an autoimmune disorder, low titres of antibody (≤1.80) with no signs or symptoms of disease are generally a non-specific finding, more common in women and elderly, as well as in patients with organ-specific autoimmune diseases (19).

This study confirms that ANA positivity is not uncommon in women with unexplained RM, suggesting the possible role of an autoimmune disorder on abortion, at least in a subgroup of patients. Our results showed that ANA positivity was generally found with a moderate-positive result (1.160-1.320) in about 46% of positive cases, whereas low titres (1.40-1.80) and high titres were seen in about 38% and 16% of cases respectively. Moderate and higher titres probably are associated with known autoimmune disorders. In the other hand diagnosis of type of autoimmune disorders is feasible by using interpretation of microscopic ANA patterns. Correlation of these patterns and autoimmune disease was mentioned in table I. This study showed that in unexplained recurrent pregnancy loss, the most common antinuclear autoantibodies were anti-dsDNA/anti-histones antibodies (homogenous pattern) associated with SLE, a systemic and multisymptomatic disease, and that should be considered in these patients based on our study.

**Table I:** Antinuclear antibodies patterns and associated disease

**ANA pattern**	**Associated antigen**	**Associated disease**
Homogenous	DNA histone complex (ribonucleoprotein, nucleosome)	SLE, drug-induced lupus, other diseases
Peripheral/rim	DNA, nuclear envelope antigens	SLE, autoimmune hepatitis
Speckled	Smith, RNP, SS-A, SS-B, Scl-70, centromere, RNA polymerase II and III	SLE, MCTD, Sjӧgren syndrome, PSS, Other disease
Nucleolar	Nucleolar RNA, RNA polymerase I	Diffuse systemic sclerosis, hepatocellular carcinoma
Centromeric	Centromer	CREST syndrom

RNP: ribonucleoproteins, SLE: systemic lupus erythematosus, MCTD: mixed connective tissue disease, PSS: primary systemic sclerosis

## Conflict of interest

There is no conflict of interest in this study. 
